# New Concepts in Therapeutic Manipulation of HIV-1 Transcription and Latency: Latency Reversal versus Latency Prevention

**DOI:** 10.3390/v15081677

**Published:** 2023-07-31

**Authors:** Catherine A. Lewis, David M. Margolis, Edward P. Browne

**Affiliations:** 1 University of North Carolina HIV Cure Center, UNC Chapel Hill School of Medicine, University of North Carolina at Chapel Hill, Chapel Hill, NC 27599, USA; catlewis@email.unc.edu; 2 Department of Microbiology and Immunology, UNC Chapel Hill School of Medicine, University of North Carolina at Chapel Hill, Chapel Hill, NC 27599, USA; 3 Division of Infectious Diseases, Department of Medicine, UNC Chapel Hill School of Medicine, University of North Carolina at Chapel Hill, Chapel Hill, NC 27599, USA; 4 Department of Epidemiology, Gillings School of Global Public Health, University of North Carolina at Chapel Hill, Chapel Hill, NC 27599, USA

**Keywords:** HIV-1 latency and reactivation, HIV-1 latent reservoir, chromatin and epigenetics, viral and host transcriptional regulators, mechanisms and therapeutic opportunities, HIV-1 eradication and cure

## Abstract

Antiretroviral therapy (ART) has dramatically improved the prognosis for people living with HIV-1, but a cure remains elusive. The largest barrier to a cure is the presence of a long-lived latent reservoir that persists within a heterogenous mix of cell types and anatomical compartments. Efforts to eradicate the latent reservoir have primarily focused on latency reversal strategies. However, new work has demonstrated that the majority of the long-lived latent reservoir is established near the time of ART initiation, suggesting that it may be possible to pair an intervention with ART initiation to prevent the formation of a sizable fraction of the latent reservoir. Subsequent treatment with latency reversal agents, in combination with immune clearance agents, may then be a more tractable strategy for fully clearing the latent reservoir in people newly initiating ART. Here, we summarize molecular mechanisms of latency establishment and maintenance, ongoing efforts to develop effective latency reversal agents, and newer efforts to design latency prevention agents. An improved understanding of the molecular mechanisms involved in both the establishment and maintenance of latency will aid in the development of new latency prevention and reversal approaches to ultimately eradicate the latent reservoir.

## 1. Introduction

Since the introduction of combination antiretroviral therapy (ART) in the 1990s, the prognosis for people living with human immunodeficiency virus 1 (HIV-1) has improved dramatically. Nevertheless, in 2021 alone, more than 1.5 million people were newly infected, and 650,000 died of causes related to acquired immunodeficiency syndrome (AIDS) [1]. While the continued spread of HIV-1 can largely be attributed to problems of access to therapy and the stigma that still accompanies an HIV-1 diagnosis, ART itself also carries with it the burden of lifelong access and adherence to therapy. Further, although ART effectively targets actively replicating viruses, HIV-1 persists as a latent reservoir of stably integrated provirus that rapidly rebounds if ART is paused [2,3,4]. Treatments that can either eradicate or permanently, reliably silence the latent reservoir would therefore offer significant benefits.

To achieve eradication, one long-standing approach seeks to develop ***latency reversal agents*** (LRAs) that target host factors involved in the regulation of proviral expression to reignite viral gene expression such that the immune system can detect and clear infected cells. Thus far, numerous LRAs capable of reactivating viral transcription in vitro or ex vivo have been identified, some of which have demonstrated the ability to reverse latency in vivo [5,6,7,8]. However, in practice, latency reversal alone does not appear to lead to the clearance of infected cells or a subsequent reduction in reservoir size. More recent clinical experiments have therefore sought to pair LRAs with immune clearance agents or immunotherapies. Thus far, however, none of the trials testing combinations of LRAs with these therapies have resulted in substantial depletion of persistently infected cells [9]. It is unclear whether the current lack of success of this approach is due to an insufficient effect of LRAs on the small and diffuse latent reservoir in either the extent or duration of viral antigen presentation or in the breadth, potency, or timing of the antiviral immune response. It is also possible that both arms of this dual strategy are flawed.

**Figure 1:**
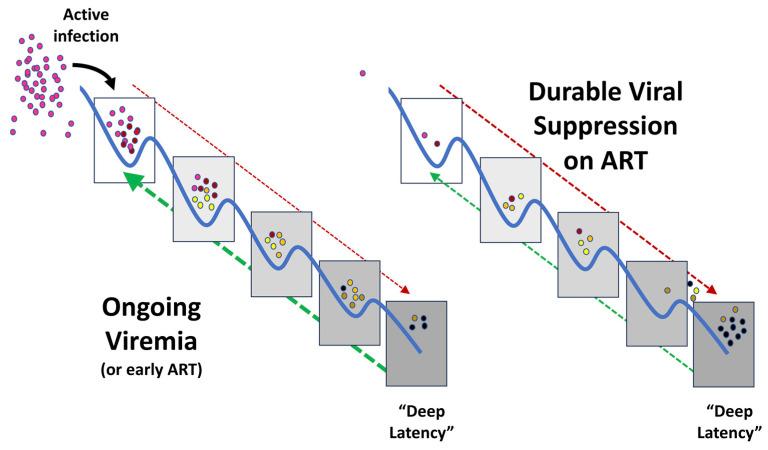
During ongoing viremia or early ART, cells are infected with circulating viral species (purple). Some of these infected cells may survive to enter gradually deeper states of proviral quiescence and latency (red arrow). Recent and older clones (illustrated by progressively darker colors) may proliferate and may enter deeper states of latency, or may progressively leave quiescence (green arrow) due to the effects of host cell or viral activation. Infected cells may also die due to immune clearance, viral cytopathic effects, or simply host cell senescence. The frequency of cells harboring the virus in “deep latency” (darker-colored circles) is therefore limited. Once ART is initiated, new infections are prevented. With the resolution of viremia, immune activation wanes, and the forces that enforce latency (red line) may strengthen, while those that induce viral expression and the exit from latency (green line) wane. Over time the frequency of latent infection reaches a balance, where the proliferation rate of latent clones is roughly matched by clonal clearance or cell death.

**Figure 2:**
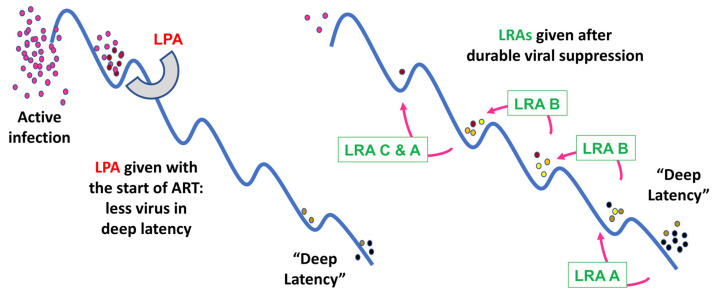
Prior to ART initiation, very little virus is in deep latency because of immune activation, host cell turnover, and higher rates of viral reactivation. Administration of a latency prevention agent (LPA) near the time of ART initiation may therefore prevent the host changes that establish and enforce latency. After ART is initiated, the latent reservoir is stabilized and subject to multiple molecular mechanisms of latency. Virus in deep latency, in particular, is suppressed by layers of host-driven mechanisms, including epigenetic modifications. A single LRA may therefore move the virus “uphill” into a shallower state of latency (red arrows) without restoring viral gene expression. A combination of distinct LRAs may be required to allow broad and clinically effective reversal of latency, making the proviral reservoir vulnerable to immune mechanisms and therapeutics to eradicate persistent HIV infection.

Recently, new findings have suggested a novel additional or alternative approach: ***latency prevention***. Whereas historically, the long-lived latent reservoir was thought to be seeded continuously beginning immediately after infection, several recent observations by independent groups have now found evidence that, although latency is established immediately after infection, much of the long-lived latent reservoir originates from virus circulating in the year before ART initiation [10,11,12] (Figure 1). This finding suggests that the latent reservoir is somewhat dynamic during viremia and that, because ART blocks new infection, blunts immune activation, and allows immune reconstitution, the resultant biological changes also favor the entry into and persistence of recently integrated proviruses in the latent state. This model suggests that intervention near the time of therapy initiation could prevent much of the reservoir formation and reduce the size of the latent pool that remains suppressed by ART [10,11,12] (Figure 2).

Unlike our developing understanding of the maintenance of latency, the epigenetic, cellular, and immunologic factors that regulate the foundation of latency have yet to be elucidated. However, it is probable that some mechanisms that can be targeted to reverse latency may also be targeted to prevent the provirus from entering the latent state. As a result, some LRA approaches might also be re-evaluated as latency prevention agents (LPAs).

This review will briefly summarize major strategies to reverse latency and discuss emerging research and potential approaches to preventing the formation of the latent reservoir. Given the nearly 30 million people already on ART and the additional 1.5 million people who are newly diagnosed with HIV-1 infection each year, both strategies may play a role in future strategies to eradicate HIV-1 infection. Latency-prevention strategies to reduce the enforcement of latency when ART is initiated may be a valuable addition to ART for people who are diagnosed and treated early in infection or following chronic HIV infection. Given that “old” viruses that have established latency during viremia will persist, latency reversal strategies, should they be possible in the future, will also likely continue to be an important component of strategies to eradicate infection.

## 2. Viral Latency: Where and How the Virus Persists

HIV-1 primarily infects activated CD4^+^ T cells as well as macrophages [2,4,13,14,15]. During acute infection, viremia rapidly increases and CD4^+^ T cell numbers decline, both as a direct result of infection and cytotoxic T cell killing and as an indirect result of immune hyperactivation and bystander killing. Once ART is initiated, active viral replication is halted. However, a small latent reservoir persists, formally defined as the pool of HIV-1-infected cells that, prior to treatment with an activating agent or LRA, do not transmit the virus to uninfected target cells in culture but can transmit the virus following such treatment. The latent reservoir represents the most formidable barrier to HIV-1 cure. A latent virus that is transcriptionally silent does not express viral proteins and is therefore difficult to target therapeutically. In addition, much of the latent reservoir is long-lived and self-renewing, with rates of natural decay too slow to allow viral clearance within the lifetime of most people living with HIV (PLWH). The eradication of the latent reservoir will therefore require specific, targeted approaches.

CD4^+^ T cell populations are the best-characterized constituent of the latent reservoir [15,16]. Central memory CD4^+^ T cells that lack activation markers (resting) in particular are the most extensively documented to harbor persistent infection [17,18]. Latency has also been demonstrated in naïve, stem cell memory, and transitional CD4^+^ T cells [19,20,21,22,23,24], but the frequency of persistent infection in these cell types appears to be lower, and these infected populations have not been longitudinally studied [22]. Although HIV genomes can be found in effector memory populations, the typically short lifespan of these cells suggests that these latently infected populations may emanate from the proliferation and differentiation of latently infected CD4^+^ T cell central memory populations [25].

Myeloid cells also likely constitute a viral reservoir, potentially contributing to viral rebound upon treatment interruption [26]. Macrophages, in particular, may be contributors, given that they are infected in vivo and have a long half-life. A new viral outgrowth assay developed specifically for monocyte-derived macrophages recently provided direct evidence that macrophages play a role in maintaining the latent reservoir [27]. Another recent study rigorously documented the recovery of latently infected microglia from the brain tissue of altruistic donors suppressed on ART until the time of death from non-AIDS causes [28]. Finally, various anatomical compartments have been reported to harbor viral RNA and/or DNA, including gut-associated lymphoid tissue, the central nervous system, the genital tract, and lymph nodes [29,30,31,32,33]. Effectively targeting the latent reservoir will require elucidating the mechanisms that establish and maintain viral latency in these different cell types and compartments.

## 3. Establishment of the Latent Reservoir

The mechanism by which the long-lived latent reservoir is established is incompletely understood. Given the permissiveness of activated CD4^+^ T cells to HIV-1 infection, one important source is the small surviving fraction of activated CD4^+^ T cells that return to rest and become long-lived memory cells following infection [34]. These latently infected CD4^+^ T cells are maintained via clonal expansion supported by several mechanisms, including the IL-7- and IL-15-driven homeostatic proliferation of memory CD4^+^ T cells, antigen-driven proliferation, and integration site-driven aberrant proliferation [21,35,36,37,38]. The relative contributions of homeostatic versus antigen-driven proliferation may vary over the course of infection, whereas the contribution of integration site-driven aberrant proliferation appears to be generally less significant [39].

The direct infection of resting or minimally activated CD4^+^ T cells to the latent reservoir may also play a role, particularly in very early, acute infection, but its importance later in infection is less clear. Because of a SAMHD1-mediated block in reverse transcription, among other factors, reverse transcription and subsequent integration is relatively inefficient in resting CD4^+^ T cells [40]. Truncated double-stranded DNA products can form one- or two-LTR circles and are never integrated. In addition, the slow rate of reverse transcription in resting CD4^+^ T cells means the majority of the full-length linear double-stranded DNA products produced are degraded before reverse transcription is completed [41]. However, infected resting CD4^+^ T cells can be induced to release the virus upon activation, demonstrating that HIV can transiently exist in a state of pre-integration latency within resting CD4^+^ T cells [41,42,43]. Resting CD4^+^ T cells can also be infected via cell-to-cell transmission, which may preferentially result in the immediate establishment of latency [44]. Therefore, latency can be established in resting CD4^+^ T cells both via cell-free and cell-to-cell transmission.

The dynamics of latency establishment, particularly in CD4^+^ T cells, are the subject of significant debate. Until recently, the prevailing model was that HIV-1 enters the latent reservoir soon after infection, supported by studies demonstrating that the latent reservoir forms even when ART is initiated immediately [4,6,45]. Efforts to eradicate the latent reservoir have therefore focused on latency reversal rather than prevention. In recent years, however, several studies have shown that the majority of the long-lived latent reservoir is most closely related to viruses circulating around the time of ART initiation, challenging the existing model of latency establishment [10,11,12].

Although it has yet to be studied directly, we have previously proposed a mechanism by which ART may alter the rate of viral entry into and exit from latency [46]. Briefly, before ART is initiated, entry and exit occur at a relatively constant rate. Entry is driven primarily by the infection of new cells, while exit is caused by immune activation that results in either virion production coupled with immune clearance and/or cell death. The latent reservoir is therefore seeded continuously via recently infected cells, but these cells persist in latency for a limited time. Upon ART initiation, new infection and therefore entry into latency is blocked, but exit is also decreased by the mechanisms that establish and maintain latency discussed in the following sections (Figure 1).

An alternative explanation for this phenomenon, suggested by Coffin and Hughes, is that ART blocks additional infections of the same cell, i.e., superinfection. Prior to ART, the small fraction of cells that survive infection likely do not express viral genes and are especially sensitive to a second infection, which causes them to die. Once ART is initiated, however, superinfection is halted, enabling the fraction of latently infected cells that have not yet been multiply infected to persist [47].

## 4. Molecular Mechanisms of Latency

HIV-1 latency, which is characterized by little to no viral gene transcription, is maintained by multiple layers of transcriptional and epigenetic control at the viral promoter (long terminal repeat, LTR). At the level of transcription, latency is maintained in resting cells by a lack of the host and viral transcription factors required to overcome promoter-proximal pausing, a mechanism that enables genes to remain poised for rapid gene expression via RNA polymerase II (RNAP II) accumulation near the transcription start site, and, in the case of HIV, at several sites downstream. In resting cells, two transcription factors required for HIV transcription initiation, nuclear factor κB (NF-κB) and nuclear factor of activated T cells (NFAT), are sequestered in the cytoplasm. Other transcription factors important for HIV-1 transcription, such as activating protein-1 which synergizes with NF-κB, are expressed at insufficient levels in resting cells to activate viral transcription [48]. Perhaps most importantly, signaling through positive transcription elongation factor b (p-TEFb), a cellular transcription factor critical for the processive elongation of viral transcription, is disrupted. In the absence of activity by these host transcription factors, the production of viral trans-activator of transcription (Tat) protein is also low, and the assembled RNAP II initiation complexes either remain paused or prematurely terminate transcription. Increased Tat expression, upon T cell activation, for example, can overcome these host restrictions by mediating the recruitment of P-TEFb and other elongation factors [49,50,51,52].

Another transcriptional mechanism that enforces latency is transcriptional interference. For example, one group reported that when the virus integrated into actively transcribed host genes, RNAP II could read through the viral LTR and, as a result, displace the preinitiation complex on the 5′ LTR. Another group reported an orientation-dependent effect of HIV integration. When HIV-1 was integrated into the same orientation as the host gene, viral gene expression was enhanced. Conversely, when host and viral genes were convergently oriented, proviral and host gene RNAP II complexes could collide, leading to aborted viral gene expression [53,54].

Epigenetic mechanisms are also important for establishing and maintaining HIV latency. These include nucleosome positioning and the recruitment of repressive epigenetic modifications. In models of HIV-1 infection, active transcription is associated with more accessible chromatin [55], whereas during latency, viral LTR is bound by two nucleosomes, nuc-0, and nuc-1, which are heterochromatic and serve as barriers to HIV transcription. Specifically, nuc-1 is positioned immediately following the transcription start site where it blocks the release of promoter-proximal transcription complexes [55]. Reactivation can occur when host transcription factors such as NF-κB are released into the nucleus where they initiate a series of events that promote chromatin remodeling, restoring transcription factor access to the viral promoter [56]. Studies in latently infected T cell line models and CD4^+^ T cells have also suggested that the two CpG islands of the viral LTR are hypermethylated, corresponding with the repression of viral gene expression [57,58]. The in vivo contribution of DNA methylation to viral latency is currently debated, but some researchers propose that, while not required for latency establishment, DNA methylation may be important for the maintenance of “deep” latency [58].

Additional epigenetic blocks that contribute to proviral silencing include the recruitment of histone deacetylases (HDACs) and histone methyltransferases (HMTs) to the LTR, resulting in the formation of repressive chromatin structure, i.e., low levels of histone acetylation and high levels of histone methylation [52]. Indeed, the first host silencing mechanism elucidated was the cooperative recruitment of HDAC1 to the viral LTR by host transcription factors YY1 and LSF [59]. Other modifications that may parallel, augment, or even supersede acetylation, such as histone crotonylation and decrotonylation, have also been recently described [60]. Conversely, proviral reactivation is characterized by a reduction in HMTs and their corresponding methylation marks as well as increased acetylation at the LTR [52]. A recent study in primary cells also showed that histone deacetylation at the LTR may serve as a “gatekeeping” event in promoting latency establishment [61]. Therefore, the switch between an activating acetylated status and a repressive deacetylated status that enables the deposition of methyl marks is likely pivotal to the formation of the latent reservoir.

Often overlooked, post-transcriptional factors are also likely to play a role in regulating HIV latency. Viral gene expression requires the processing of viral RNA (vRNA), both during and after transcription. As with eukaryotic mRNA, this includes vRNA capping and polyadenylation as well as methylation. Additionally, HIV vRNA must undergo alternative splicing for the production of the full array of viral proteins. Finally, ribonucleoprotein complexes must be formed prior to vRNA trafficking and nuclear export. Failure to complete any of these processes will decrease viral gene expression and could result in viral latency [32]. Indeed, multiple studies have indicated that host proteins involved in RNA processing and metabolism can contribute to viral latency [62,63]. For example, a study in three primary cell models reported that latently infected cells possess a reversible block in the production of multiply spliced HIV RNA [63].

It is clear that viral latency is established and maintained by multiple factors that modulate viral gene expression and virion production, including chromatin accessibility, transcription initiation and elongation, and post-transcriptional processing and translation. We and others have reviewed mechanisms of latency in greater detail in [46,64,65]. The following sections summarize efforts to target these factors to promote viral gene expression using LRAs and discuss new approaches to targeting some of these factors to prevent latency establishment.

## 5. Strategies for Eradicating Latent HIV Reservoirs: Latency Reversing Agents

Early latency-reversing agents: The first approaches to latency reversal sought to broadly activate T cells, typically via IL-2 or anti-CD3 antibodies. However, such approaches, tested in clinical trials in the late 1990s through the early 2000s, failed to reduce the latent reservoir [66,67] and, in one case, were associated with significant toxicity [68]. As a result, latency reversal efforts were dropped for a time before a shift toward approaches that more specifically target the transcriptional and epigenetic mechanisms that suppress viral gene expression without inducing universal T cell activation. The major classes of LRAs include epigenetic modifiers (HDAC inhibitors, HMT inhibitors bromodomain extraterminal domain inhibitors), host transcriptional activators (protein kinase C agonists, NF-κB agonists), and immunomodulatory compounds (Toll-like receptor agonists, IL-15 agonists, immune checkpoint inhibitors).

Epigenetic LRAs: Significant efforts have focused on identifying compounds that can target the repressive epigenetic factors that silence viral gene expression. One of the earliest studies found that treating CD4^+^ T cells from people with HIV with small molecules that inhibit both LSF binding and HDAC1 recruitment to the viral LTR-induced viral outgrowth, demonstrating that HDAC inhibition can reverse latency [69]. This, combined with the fact that several HDACis have already been approved to treat T cell lymphomas, has led to significant interest in examining the LRA potential of HDAC inhibitors (HDACis) in HIV infection.

HDACis can be grouped into four structural classes: aliphatic acids (valproic acid, C_8_H_16_O_2_), hydroxamic acids (trichostatin A, C_17_H_22_N_2_O_3_; vorinostat, C_14_H_20_N_2_O_3_; panobinostat, C_21_H_23_N_3_O_2_), benzamides (entinostat, C_21_H_20_N_4_O_3_), and cyclic tetrapeptides and depsipeptides (trapoxin B, C_33_H_40_N_4_O_6_; romidepsin, C_24_H_36_N_4_O_6_S_2_). Many HDACis such as trichostatin A and valproic acid induce HIV transcription in latently infected cells [70]. However, when valproic acid, one of the first HDACis to be tested in vivo, was administered to patients in conjunction with highly active ART, it did not uniformly or reliably reduce the size of the latent reservoir [70,71,72,73,74]. The more potent HDACi vorinostat [6,75] has been found to reverse latency in vivo in terms of the level of cell-associated unspliced (CA-US) HIV-1 RNA in resting CD4^+^ T cells. This finding was replicated in subsequent studies, but viremia was not induced [76,77]. Similar studies with panobinostat, givinostat, and romidepsin were successful in increasing both CA-US HIV-1 RNA levels as well as viremia. However, no reduction in the frequency of latently infected cells was observed by latency reversal alone [78,79,80].

Other approaches that target the repressive proviral chromatin architecture that characterizes latency have included DNA methyltransferase inhibitors, such as 5-aza-2′-deoxycytidine, which has been shown to restore viral gene expression in memory CD4^+^ T cells isolated from PLWH [58]. Similarly, using epigallocatechin-3-gallate to inhibit ubiquitin-like with PHD and RING finger domain 1, a protein involved in both DNA and histone methylation, in primary CD4^+^ T cell models and peripheral blood mononuclear cells taken from PLWH also increased CA-US HIV-1 RNA levels to higher levels than did TCR activation [81].

Another epigenetic approach to latency reversal has been to promote either the initiation of viral transcription by inhibiting HMTs or to promote transcription elongation by inhibiting the bromodomain and extra-terminal (BET) domain family. The HMT inhibitors chaetocin and BIX-01294 have been shown to increase HIV-1 recovery from ex vivo cultures of resting CD4^+^ T cells isolated from PLWH on ART [82]. Similarly, the small-molecule BET inhibitors JQ1, RVX-208, and PF-1 activated HIV transcription in latently infected Jurkat T cells and a primary cell model [83,84,85]. Overall, HMT and BET inhibitors have shown only modest effects on latency reversal and have yet to be tested in PLWH [86].

Host transcriptional activators: NF-κB activation, via both canonical and non-canonical pathways, plays an important role in activating viral gene expression and, as a result, has attracted significant attention as a potential LRA target [87]. Five proteins comprise the NF-κB family: NF-κB 1 (p50), NF-κB 2 (p52), RelA (p65), RELB, and c-REL. Whereas the canonical NF-κB pathway rapidly and transiently activates a broad range of diverse genes, the non-canonical pathway primarily triggers NF-κB2 and RelB with slower but more persistent kinetics. Both pathways have been identified as potential LRA targets. For example, protein kinase C (PKC) agonists, which activate NF-κB through the canonical pathway, have been shown to promote viral gene expression and cell activation in vitro and in cells taken from SIV-infected macaques [88,89]. However, one phase 1 clinical trial with the PKC agonist bryostatin-1 failed to induce RNA transcription in PBMCs or viremia, and many PKC agonists have proven toxic in vivo [90]. One alternative proposed has been disulfiram, which is already used to treat chronic alcoholism and is known to promote the nuclear entry of NF-κB1 and RELA. However, studies have shown little to no effect on viral transcription and no reduction in reservoir size [91,92].

Given the narrower range of transcription factors activated and the more favorable kinetics, targeting the non-canonical NF-κB pathway may have higher specificity and less toxicity. In addition to activation via receptor ligation, the non-canonical pathway can be activated at intermediate steps in the pathway, such as via augmenting the activity of the second mitochondria-derived activator of caspases (SMAC) [93]. First developed to promote apoptosis in tumor cells, SMAC mimetics have been shown to induce gene transcription in in vitro latency models as well as in resting CD4^+^ T cells from people on ART and a bone marrow, liver, and thymus (BLT) mouse model of HIV infection [88,94,95]. Further, the SMAC mimetic AZD5582 was shown to induce viral gene expression in the blood and tissues of ART-suppressed HIV-infected BLT humanized mice and SIV-infected rhesus macaques [7]. Increased CA-RNA and viremia were also observed. Although non-canonical NF-κB pathway agonists have not yet resulted in a reduction in the latent CD4^+^ T cell reservoir, their use holds promise as a more specific approach than most other LRA candidates.

Immunomodulatory compounds: Immunomodulatory compounds have the potential dual benefit of not only promoting viral gene expression but also restoring immune function, which, together, could lead to the elimination of infected cells. In contrast to other approaches, which typically directly activate or promote viral gene expression in CD4^+^ T cells, many immunomodulatory compounds have a more indirect effect. For example, pattern recognition response ligands/agonists sense antigens leading to cytokine release, which, in turn, can activate CD4^+^ T cells. The best-studied is Toll-like receptor 7 (TLR7), which induces type I interferon production that results in CD4^+^ T cell activation. In an SIV model in ART-treated rhesus macaques, a TLR7 agonist-induced viral gene expression as well as a reduction in SIV DNA and the amount of ConA-reactivated SIV produced by PBMCs. Further, there was no viral rebound in two of nine animals following ART interruption [96]. However, these results were not reproducible by other groups [97,98], although one group reported delayed viral rebound in simian–human chimeric immunodeficiency virus-infected macaques receiving the TLR7 agonist GS-9620 in combination with the broadly neutralizing antibody PGT121 [99].

Other studies have examined the LRA potential of the TLR9 ligand MGN1703. Although an initial study found increased viremia and activation of some immune cell types, in a follow-up study by the same group, no change in CA-RNA or the size of the latent reservoir was observed [100]. TLR3 agonists have generally been unsuccessful at inducing latency reversal. Thus far, approaches that target TLR3, 7, and 9 may have largely been unsuccessful because their effects on CD4^+^ T cells are indirect [86]. In addition to studies on these extracellular pattern recognition receptors, additional work has examined the potential of cytosolic pattern recognition receptors, such as retinoic acid-inducible gene I (RIG-I)-like receptors and the RIG-1-inducible gene STING, which activates NF-κB, among other factors. The FDA-approved retinoic acid derivative acitretin has been shown to increase HIV transcription in vitro and to induce apoptosis in HIV-infected cells [101]. A study on STING ligands 2′3′-cGAMP and c-d-AMP showed that these ligands could increase SIV RNA levels and decrease DNA levels in PBMCs isolated from cynomolgus macaques [102].

Another immunomodulatory approach to latency reversal is to target immune checkpoint inhibitors (ICIs). Chronic HIV infection is associated with immune dysregulation, including the overexpression of co-inhibitory receptors, such as PD-1, CTLA4, LAG3, TIGIT, and Tim3, and the progressive exhaustion of HIV-specific CD8^+^ T cell [103]. One proposed approach to latency reversal has therefore been to block co-inhibitory receptors, with the dual goals of reversing latency and restoring the immune function of exhausted HIV-specific T cells.

Because ICIs were developed primarily for malignancies, most LRA candidates were initially identified in case studies of people with HIV and cancer. Some examples include anti-CTLA4 Ab (Ipilimumab), which resulted in increased CA-RNA and, surprisingly, decreased viremia in a person with HIV and melanoma. Anti-PD1 (nivolumab) in the same patient resulted in increased CA-RNA but no change in plasma HIV RNA [104]. In a preclinical study on ART-suppressed SIV-infected rhesus macaques, treatment with monoclonal-antibody targeting PD-1 or CTLA4 led to an increase in viral gene transcription and viremia and a decrease in intact provirus [105]. However, significant autoimmunity-related adverse effects were observed in a trial of anti-PD1, leading to the early termination of the trial [106].

The persistent immune activation caused by chronic infection can also lead to immunosenescence. Senotherapeutic compounds, which target senescent cells, may therefore also be good LRA candidates. For example, one study showed that an mTOR inhibitor could reduce the production of the pro-inflammatory cytokines induced by anti-TCR stimulation. Similarly, the combination of the mTOR inhibitor and the PKC agonist bryostatin-1 could inhibit cytokine release [107]. The same effect was observed in a separate study using a Janus kinase inhibitor and the PKC agonist ingenol B [108]. Taken together, these studies suggest that the addition of mTOR or Janus kinase inhibitors to treatment with T cell activators may reduce toxicity, thereby restoring the possibility of using LRAs that broadly activate T cells.

Additional immunomodulatory approaches include stimulation with IL-7 or IL-15 to boost cellular immunity with less toxicity than IL-2. One study in HIV-infected humanized mice stimulated ex vivo in the presence of ART showed that IL-7 induces p24 expression from thymocytes and splenocytes [109]. However, a multicenter clinical trial assessing the effect of ART intensification followed by IL-7 administration reported only a transient increase in CA-RNA and no reduction in the latent reservoir [110]. A phase 1 clinical trial with an IL-15 superagonist (N-803) reported increased HIV transcription in memory CD4^+^ T cells but also showed evidence of the proliferation of infected cells [111].

## 6. Latency Prevention Agents: A New Concept

Whereas latency reversal and LRAs have been subjects of significant study, the concept of latency prevention has emerged only in recent years, based on studies indicating that the majority of the circulating latent reservoir is seeded around the time of ART initiation [10,11]. The mechanisms that regulate entry into latency are therefore not well understood. However, because the provirus that is actively being transcribed is not yet subject to the multiple layers of transcriptional and epigenetic silencing that are important for maintaining viral latency, latency prevention may be more efficacious than latency reversal in vivo. Our current model suggests that recently established proviruses are sensitive to the potential effects of latency prevention strategies employed close to the time of ART initiation, and so it is hoped that time-limited interventions to prevent the enforcement of viral latency at the time of ART initiation could be paired with the clinical monitoring employed as standard practice for ART initiation (Figure 2). Several approaches have been proposed based on new research on latency formation in a primary CD4^+^ T cell model and data from latency reversal studies.

Targeting proviral epigenetic mechanisms: In a recent study, we reported that, in a primary CD4^+^ T cell model of HIV-1 latency treated with a panel of epigenetic inhibitors, only class I HDACis maintained viral gene expression. Persistent viral gene expression was accompanied by elevated H3K9 acetylation and reduced H3K9 methylation at the viral promoter region [61]. We concluded from these results that HDACs may serve as gatekeepers to latency; by removing acetyl groups from histone tails, HDACs clear the way for the deposition of repressive epigenetic modifications that promote viral entry into deeper states of latency. Further, we observed that HDAC inhibition with vorinostat affected effector-to-memory transition, promoting entry into shorter-lived, more highly differentiated memory CD4^+^ T cell subsets, indicating that HDACs may also be important for maintaining CD4^+^ T cells in a longer-lived, more pluripotent state, characteristics of cells that harbor the long-lived latent reservoir [21,61]. Based on this study in a primary cell model of latency prevention, we proposed that class I HDAC inhibitors may be effective at reducing the size of the latent reservoir when administered around the time of ART initiation.

Two clinical trials have administered an HDACi with the initiation of ART, both in the context of recent or acute HIV infection [112]. While both romidepsin and vorinostat were well-tolerated, both studies employed multiple interventions, so neither was able to document the effect of an HDACi on the frequency of latent infection over time. A clinical trial to test the effect of two weeks of vorinostat on the frequency of latent infection when added to initial ART is in development in the NIH AIDS Clinical Trials Group.

Targeting CD4 T cell biology: IL-7/IL-7R-signaling block: A second proposed approach to latency prevention is based on the observation that acute HIV infection skews memory CD4^+^ T cells toward shorter-lived effector memory cells and reduces the frequency of long-lived cells, whereas ART initiation restores the effector-to-memory transition and the maintenance of long-lived cells. During untreated HIV-1 infection, immune function is highly dysregulated, with ongoing viral replication stimulating immune activation. This generalized immune activation also leads to a decrease in the formation of long-lived memory CD4^+^ T cells, likely due at least in part to dysregulated IL-7/IL-7R signaling. Following ART initiation, viremia and immune activation dramatically decrease, restoring near-homeostatic levels of IL-7/IL-7R. The authors propose that during ART initiation, the effector-to-memory transition is restored as a result, leading to an increase in the pool of long-lived memory cells harboring the latent virus. This long-lived latent reservoir is, in turn, maintained by homeostatic proliferation also induced by the restored IL-7/IL-7R signaling. Therefore, IL-7/IL-7R signaling blockade during ART initiation could be a tractable strategy to reduce the size of the long-lived latent reservoir within CD4^+^ T cells [113]. The feasibility of this strategy is further supported by the fact that monoclonal antibodies that antagonize IL-7R are already under investigation for other diseases and appear to be well-tolerated in healthy individuals [114].

## 7. Therapeutic Challenges

Challenges for LRAs: Latency reversal efforts face significant challenges, largely due to the heterogeneity of the cells and compartments that harbor the latent reservoir. First, each compartment presents unique pharmacologic challenges in terms of LRA delivery. In addition, it is unlikely that a single approach will be effective at targeting viral latency in multiple cell types and compartments. Even within the different CD4^+^ T cell subsets, LRAs have been shown to induce varying degrees of viral gene expression [115,116]. This variation is likely to increase still further when comparing the effects of an LRA on CD4^+^ T cells versus microglial cells, for example. Combinations of agents may also be required to target the complex mechanisms involved in maintaining transcriptional silencing. Indeed, multiple studies have shown a synergistic effect when combining LRAs [117]. Finally, given that some LRAs have moderate latency reversal activity but none alone have been effective at reducing the size of the latent reservoir, it is likely that LRAs will need to be combined with immunotherapies that facilitate the clearance of infected cells. The combination of an LRA with an immune clearance agent has been tested in multiple recent clinical trials but has not yet seen success in terms of reducing the size of the latent reservoir [118,119,120].

Challenges for LPAs: LPA studies will likely encounter some of the same challenges that LRAs have faced, namely that multiple approaches may need to be taken to target the varied mechanisms involved in the formation of the latent reservoir in addition to the many cell types and compartments involved. Unique to LPA strategies is the caveat that the proposed latency prevention strategies will not target the fraction of the latent reservoir that already persists in long-lived cells. In addition, the studies demonstrating that most of the latent reservoir forms around the time of ART initiation were performed in peripheral CD4^+^ T cells. However, the question of whether the latent reservoir is seeded with similar dynamics in other cell types and tissues is currently unclear and merits additional study.

## 8. Summary/Conclusions

Significant progress has been made in understanding the mechanisms involved in maintaining HIV latency, particularly in CD4^+^ T cells. As a result, strategies with moderate efficacy in restoring viral gene expression and viremia have been identified not only in animal models but also in clinical trials. However, at present, none have been successful in reducing the size of the latent reservoir. Ongoing research is therefore focused on examining both combinations of LRAs to target the multiple layers of regulation that maintain viral transcriptional silencing and the multiple cell types and compartments involved as well as the combination of LRAs with immune clearance agents.

Newer research has suggested that it may also be possible to prevent the formation of a significant fraction of the long-lived latent reservoir, thereby reducing the size of the inducible latent reservoir that would require targeting by LRAs. This approach would require first administering an LPA as soon as ART is initiated, followed by ongoing treatment with LRAs and immune clearance agents to fully eliminate the inducible latent reservoir. To achieve this goal, significant work will need to be done, particularly in animal models, to better understand the dynamics of reservoir seeding in different compartments around the time of ART initiation. 

Moving forward, new observational tools that enable the study of infected cells at the single-cell level will facilitate a better understanding of latency establishment and maintenance in different cell types and compartments. More fully elucidating the mechanisms involved in latency will facilitate the identification and assessment of new LRAs and LPAs that can decrease the size of the latent reservoir and, ultimately, lead to a cure for HIV-1.
